# Men’s reproductive coercion of women: prevalence, experiences, and coping strategies—a mixed method study in urban health facilities in León, Nicaragua

**DOI:** 10.1186/s12905-021-01441-y

**Published:** 2021-08-23

**Authors:** Cecilia Brenner, William J. Ugarte, Ida Carlsson, Mariano Salazar

**Affiliations:** 1 Regional Office of Communicable Diseases, Uppsala, Uppsala Region Sweden; 2 Department of Women’s and Children’s Health, International Maternal and Reproductive Health and Migration Research Group, Uppsala University, Uppsala, Sweden; 3 Doctors of the World/Médecins du Monde, Stockholm, Sweden; 4 Department of Global Public Health, Global and Sexual Health Research Group, Karolinska Institutet, Tomtebodavägen 18a, Widerströmska Huset, 171 77 Stockholm, Sweden

**Keywords:** Reproductive coercion, Contraceptive sabotage, Pregnancy promotion, Coping strategies, Mixed-methods

## Abstract

**Background:**

Reproductive coercion (RC) is a common form of violence against women. It can take several expressions aiming at limiting women’s reproductive autonomy. Thus, the frequency and how reproductive coercion can be resisted must be investigated. There is limited research regarding RC in Latin America. Therefore, this study aimed to measure RC prevalence and associated factors and to explore the women experiences and coping strategies for RC.

**Methods:**

A convergent mixed-methods study with parallel sampling was conducted in Nicaragua. A quantitative phase was applied with 390 women 18–35 years old attending three main urban primary health care facilities. Lifetime and 12 months of exposure to RC behaviors including pregnancy promotion (PP) and contraceptive sabotage (CS) were assessed. Poisson regression with a robust variance estimator was used to obtain adjusted prevalence rate ratios and 95% Confidence Intervals (CIs). In addition, seven in-depth interviews were collected and analyzed using qualitative content analysis.

**Results:**

Ever RC prevalence was 17.4% (95% CI, 13.8–21.6) with similar proportions reporting ever experiencing PP (12.6%, 95% CI 9.4–16.3) or ever experiencing CS (11.8%, 95% CI 8.7–15.4). The prevalence of last twelve months RC was slightly lower (12.3%, 95% CI, 9.2–16.0) than above. Twelve months PP (7.4%, 95% CI 5.0–10.5) and CS (8.7%, 95% CI 6.1–12.0) were also similar. Women’s higher education was a protective factor against ever and 12 months of exposure to any RC behaviors by a current or former partner. Informants described a broad spectrum of coping strategies during and after exposure to RC. However, these rarely succeeded in preventing unintended pregnancies or regaining women’s long-term fertility autonomy.

**Conclusions:**

Our facility-based study showed that men’s RC is a continuous phenomenon that can be enacted through explicit or subtle behaviors. Women in our study used different strategies to cope with RC but rarely succeeded in preventing unintended pregnancies or regaining their long-term fertility autonomy. Population-based studies are needed assess this phenomenon in a larger sample. The Nicaraguan health system should screen for RC and develop policies to protect women’s reproductive autonomy.

**Supplementary Information:**

The online version contains supplementary material available at 10.1186/s12905-021-01441-y.

## Background

Reproductive Coercion (RC) is one of the many forms of violence against women (VAW) and constitutes a set of behaviors aiming at limiting a woman’s reproductive autonomy [1]. These controlling behaviors can range from forcing a woman to become pregnant (either verbally, physically, or by sabotaging her contraceptives) to limit her access to elective abortion services where those services are legal. In addition, it can include forcing a woman to terminate a pregnancy that she wants to continue [1].

Reproductive coercion of women is quite common [1–3] and can be exerted by current or previous partners and/or other family members. The prevalence varies between settings [2–4], ranging from 20% to 19% among women attending health facilities in Jordan [5] and in the USA [2], 18.5% in rural Cote d’Ivoire [6] to 12% among currently married women in Uttar Pradesh, India [7]. Reproductive coercion can take several expressions such as disapproval of the woman’s contraceptive usage, interfering with the woman’s usage of contraceptives and male partner refusing to use contraceptives during sexual intercourse [2, 5, 6, 8, 9].

Like other expressions of violence, RC does not occur in a vacuum as it is influenced by social and individual factors in a given setting. Demographic factors such as women’s age, women’s low socioeconomic status, and parity have been reported as RC risk factors [10]. However, other studies have found that age was the only factor increasing RC exposure [11]. Women’s education has also been reported as a risk or protective factors across settings [12].

Women’s exposure to emotional, physical, and sexual intimate partner violence (IPV), has also been consistently shown to be a key risk factor for RC exposure [1–3, 13–15]. Although RC is an expression of IPV in itself, several studies show a higher risk of exposure to RC among women who experience other forms of IPV. Nevertheless, since RC also has been found to exist in relations with no other expressions of IPV [3], we believe that it needs to be studied as a separate phenomenon. Endorsement of unequal societal gender norms such as male dominance and control over women has also been associated with a higher risk of RC [2, 3, 14, 16, 17].

Men’s reproductive coercion of women has been associated with several negative health outcomes such as unintended pregnancies, sexually transmitted infections (STI), miscarriages and pregnancy complications [10, 15, 18]. Reproductive coercion can also have a lasting socioeconomic impact on women’s lives, since a lack of access to contraception and not being able to freely decide over ones’ reproduction can curtail women’s access to education and the benefits that it brings [10, 11, 19]. Exposure to men’s RC might be one of the factors contributing to the high unintended pregnancy rates in Latin America [20].

Studies have shown that women cope with men’s RC in different ways [12]. These might include hiding contraceptives from partners [6, 21], using a contraceptive method that they can control (i.e. an intrauterine device [1, 21] and having abortions (safe or unsafe depending on the country) among others [1, 6, 21, 22].

In this study, we used a theoretical framework where the different expressions of RC are structured in timely relation to a sexual act and expressed by an intimate partner [1].

Before sex, RC can be expressed via *pregnancy promotion*, which means that the man in different ways is expressing a wish to have a child, regardless of what the woman wants or feels. Pregnancy coercion can include threats or aggression. RC before sexual intercourse can also be expressed via *contraceptive sabotage*, for example, destroying or throwing away birth control pills [1].

Men’s RC can be manifested during the sexual act through forced sexual interaction as well as through contraceptive sabotage like refusing to withdraw if that’s the method agreed upon. Examples of condom manipulation have also been identified, where the male partner has been making holes in the condom. Additionally, RC can be expressed post-contraception, through control of pregnancy outcomes, like pressuring the woman to not have an abortion. The post-contraception RC has also been seen to be expressed through interference with healthcare like obstructing appointments for abortion [1].

Nicaragua is patriarchal society that in the last 40 years has experienced significant gains and losses in women’s empowerment, autonomy, and agency. During the 1990’ a strong civil society lead a series of legislative changes that resulted in better laws and public services for women exposed to VAW and rose awareness about it [23]. However, in the last 15 years these gains have been slowly lost due governmental policy changes [23]. For example, Nicaraguan women with unintended pregnancies have no access to safe abortion services since elective and therapeutic abortions have been banned in the country since 2006 [24] a fact that forces them to continue with the pregnancy or use unsafe abortion providers.

In spite of high contraceptive use prevalence [25], data have shown that Nicaragua has high teenage (83 births per 1000 women aged 15–19 years)[26] and unintended pregnancy rates (37% among ever partnered women)[27]. Exposure to different forms of IPV has been shown to be a key determinant of poor reproductive health in this setting [27]. For example, exposure to controlling behavior by a current or former partner (i.e. limiting a woman’s contact with family, etc.) increased women’s odds of having an unintended pregnancy by 26% [27].

Male RC of women has been found to be an important global barrier undermining women’s reproductive autonomy and health [17]. In spite of its relevance, studies using a comprehensive methodology to accurately measure it have been conducted mainly in high-income settings [1–3, 13] which limits their generalizability to low-and middle-income countries. In addition, most of the aforementioned studies have not explored how women cope (if any) with men’s RC. In Latin America, there are no studies measuring men’s RC of women using a comprehensive behavioral-based methodology. This is especially relevant for this region since unintended pregnancies rates are higher than the average for low-income settings (96 vs. 65 per 1000 women aged 15–44 years) [28] and RC could be one factor behind these figures.

Thus, we aim to fill this knowledge gap by conducting a mixed-method (MM) research design combining a quantitative facility-based survey to measure RC prevalence and associated factors with a qualitative study exploring how women experience and cope with RC. Our MM research question aims to understand how our qualitative data collection can confirm and enhance our quantitative findings. Our study will be the first in Latin America to analyze this phenomenon using a comprehensive MM approach.

## Methods

**Fig. 1:**
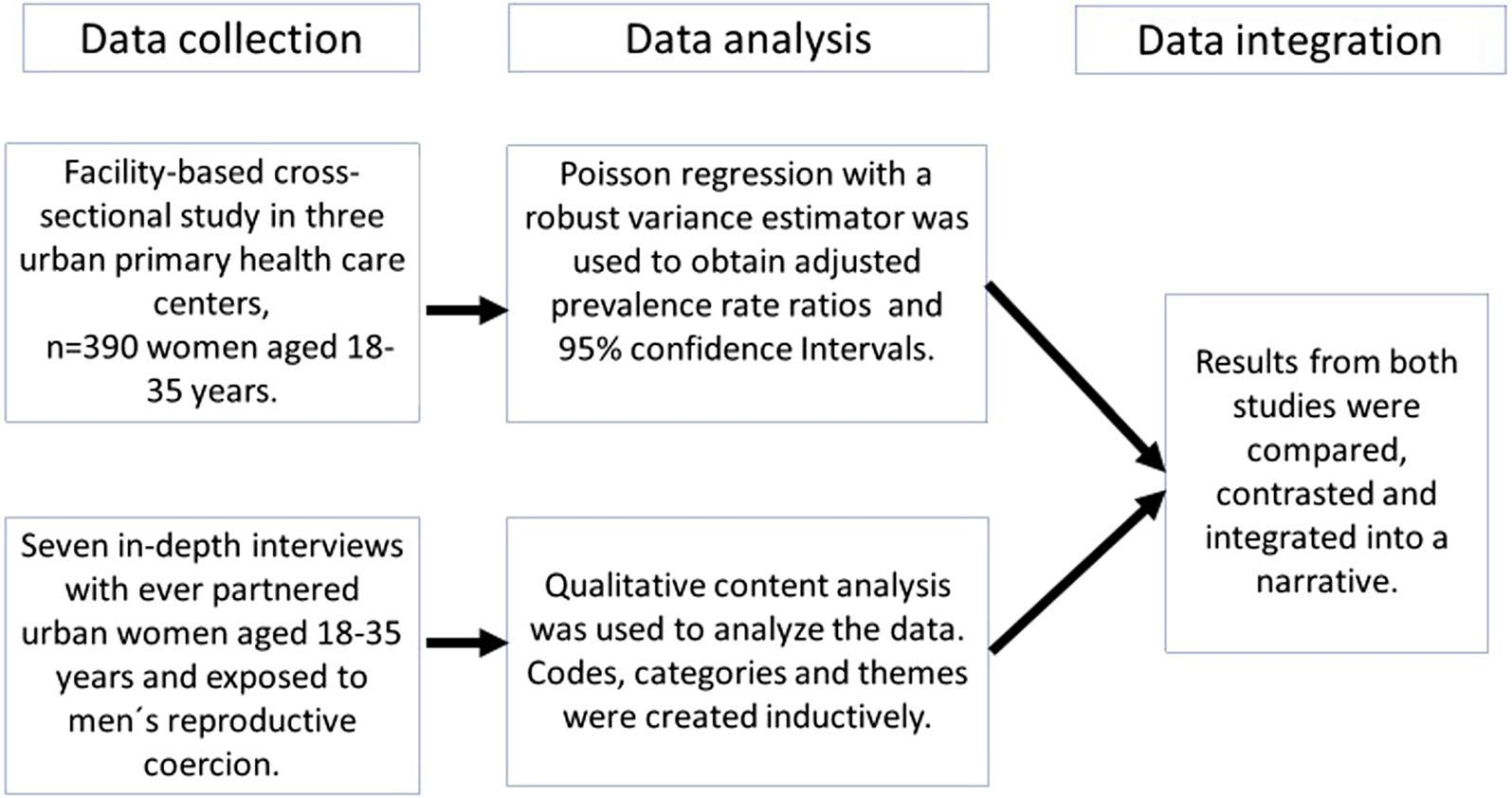
Convergent mixed-method design with parallel sampling used in the study

A convergent mixed-method design with parallel sampling [29] was used to collect and analyze the data. With this design, qualitative and quantitative data were collected in parallel; the data were analyzed separately and then merged into one result section (Fig. 1).

### Quantitative study

#### Study design and sample size

A cross-sectional facilitate based study was conducted in three main urban primary health care (PHC) centers in León city, Nicaragua. Women aged 18–35 years old, irrespective of the reason for care-seeking, were asked to participate. We focused on this age group because the impact of RC early in life can have long lasting consequences if not detected and addressed early.

A sample size of 384 was estimated using the following parameters: a. population size: one million, b. estimated RC prevalence of 20% [3, 14], c. 80% power and four % absolute precision. Consecutively, 447 women were invited and 57 declined to participate. The main reasons for declining were time constraints and the company of children or partners.

#### Variables

Lifetime and 12 months exposure to RC were assessed using a modified version of a scale developed by Silverman and colleagues [17]. The instrument measured 12 RC behaviors including pregnancy promotion (PP) and contraceptive sabotage (CS). Answering “yes” to any of the actions described below was considered RC. The same procedure applied for creating the PP and CS variables.

Items measuring PP by a current or former partner included the following: (1) threatening to leave her if not pregnant, (2) forcing her to have sex without a condom to get pregnant, (3) accusing her of being unfaithful if using birth control methods (BC), (4) threatening to be unfaithful if not pregnant, (5) threatening or physically hurting her if not pregnant and (6) pressured her to get pregnant.

Items measuring CS included: (1) removing a condom during sex, (2) refusing to give money to buy BC, (3) forbidding her to go to clinic/pharmacy/doctor for BC, (4) preventing her (with threats or blows) to go to clinic/pharmacy/doctor for BC, (5) breaking/making holes in condoms and (6) destroying BC.

The women’s sociodemographic characteristics collected included: age (years), education (none, school, secondary school, university), religion (none, catholic, protestant/evangelist, other), marital status (married, partnered, occasional partner, single), working status (housewife, student, self-employed, employed, unemployed) and pregnancy history (ever and current). Women’s last partner education (none, primary school, secondary school, university) and employment status (unemployed, student, employed) were also measured.

#### Analysis

Univariate, bivariate and multivariable analysis were conducted. Chi-square and t-test were used to compare differences between groups. Poisson regression with a robust variance estimator was used to obtain adjusted prevalence rate ratios (APRR) and 95% confidence intervals (CIs). Poisson regression was chosen over logistic regression because the latter overestimates the risks when the prevalence of the outcome is over 10% [30]. This allowed us to obtain more accurate risk estimates. Variables that were associated with the outcomes at a *p* value < 0.20 during bivariate analysis were included in the multivariable analysis. All analysis were considered significant if *p* < 0.05.

### Qualitative study

#### Participants and data collection

Ever partnered women 18–35 years old, residing in urban regions of León, and having experienced RC from a current or former intimate partner were invited to participate in the study. Purposeful heterogeneous sampling was applied to select the participants. Participants were identified from different sources including women who reported RC in the quantitative survey and through community and institutional stakeholders (NGOs and healthcare professionals).

In total, seven in-depth interviews were conducted in Spanish by the first author with the support of a local research assistant. A semi-structured interview guide was developed by the research team based on literature review and their own experience. The interview guide included vignettes, open-ended questions, follow-up and probing questions on women’s experiences with RC, reproductive decision making, and strategies used to cope with RC (if any) (please see Additional File 1 for the English version.).

Table 1 describes the informants’ characteristics. The interviewees were between 19 and 30 years old and a majority had finished or had started a university education, although a majority self-identified as housewives at the time of the interview. Two were single and all but one had children.

**Table 1:** Study population qualitative method

Informant	Age range	Occupation	Education	Number of children	Marital status	Reproductive coercion experienced
Informant no 1	20–25	Unemployed	College education	2	Single	Forced sexual relations; pregnancy promotion; contraceptive sabotage; manipulation
Informant no 2	26–30	Housewife	College education	1	Married	Control as care; violating integrity; pregnancy promotion
Informant no 3	20–25	Employed	Primary school	3	Single	Forced sexual relations; pregnancy promotion; contraceptive sabotage; undermining woman’s self-efficacy; shaming/humiliation
Informant no 4	20–25	Employed	College education	0	Partner, not living together	Threat; pregnancy promotion; manipulation
Informant no 5	26–30	Housewife	Primary school	2	Married	Control as an expression of care
Informant no 6	20–25	Housewife	College education	1	Married	Pregnancy promotion; forced sexual relation; shaming/humiliation
Informant no 7	20–25	Employed	College education	1	Partner, not living together	Withholding information

#### Analysis

The interviews were audio recorded and transcribed verbatim. Qualitative content analysis was used to analyze the data [31]. Transcripts were read first to get familiar with the data. Then, we started coding the data to identify the manifest and latent content of the text [31]. We did this process inductively meaning that we did not use predefined codes but constructed them as we were coding. Codes that had something in common were later grouped into categories. As before, these were created inductively and were not defined a priori. Finally, we contrasted and compared the categories to identify the overall meaning running through them (themes). Data were coded using OpenCode version 3.4, a freeware developed by Umeå University [32].

## Results

### Quantitative

#### Sample characteristics

The mean age among the women in the quantitative part of the study was 25.3 years (SD 4.5). Eighty-five percent had some level of secondary or university education and one in ten was single at the time of data collection. Almost half (47.2%) described themselves as housewives and 26.4% were currently pregnant. Eight in ten reported that their last partner had some level of secondary or university education. Most of the participants (46%) defined themselves as Catholics (Table 2).

**Table 2:** Women’s and their last partners’ demographic characteristics, n = 390

Characteristics	All women (n = 390)	
	n	%
Age. Mean (SD)	25.3 (4.54)	
Women’s education		
None	6	1.54
Primary school	51	13.08
Secondary school	173	44.36
University	160	41.03
Women’s religion		
None	85	21.79
Catholic	181	46.41
Protestant/evangelist	102	26.15
Other	22	5.64
Marital status		
Married	138	35.38
Partnered (not married)	177	45.38
Occasional partner	23	5.90
Single	52	13.33
Women’s working status		
Housewife	184	47.18
Student	71	18.21
Self-employed	61	15.64
Employed	50	12.82
Unemployed	24	6.15
Ever pregnant. Yes	340	87.18
Currently pregnant. Yes	103	26.41
Last partner’s education		
None	21	5.38
Primary school	48	12.31
Secondary school	177	45.38
University	144	36.92
Last partners’s working status		
Unemployed	54	13.85
Student	36	9.23
Employed	300	76.92

#### Reproductive coercion prevalence

Ever RC prevalence was 17.4% (95% CI, 13.8–21.6) with similar proportions reporting ever experiencing PP (12.6%, 95% CI 9.4–16.3) or ever experiencing CS (11.8%, 95% CI 8.7–15.4). The prevalence of last twelve months RC was slightly lower (12.3%, 95% CI, 9.2–16.0) than above. Twelve months PP (7.4%, 95% CI 5.0–10.5) and CS (8.7%, 95% CI 6.1–12.0) were also similar.

**Fig. 2:**
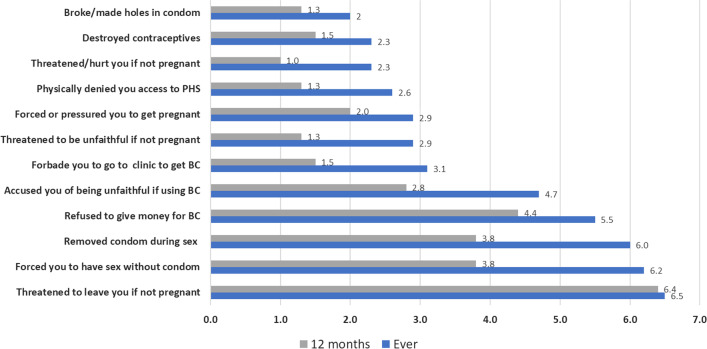
Prevalence of different forms of reproductive control, ever and last 12 months exposure, n = 390

The three most common ever RC behaviors experienced by women were threatening to leave partner if not pregnant (6.5%), forcing sex without a condom (6.2%), and removing condoms during sex (6%). Threatening to leave partner if not pregnant (6.4%), refusing to provide money for BC (4.4%), and removing condoms during sex (3.8%) were the three most common RC exposures in the last 12 months (Fig. 2).

### How was RC enacted?

The RC exercised by partners in the studied setting was seen to have different aims; to make the woman pregnant, but also to mainly control how or what kind of birth control to use. Our qualitative data allowed us to identify the pervasive and the subtle pathways by which RC behaviors were enacted. We found examples of contraceptive sabotage, pregnancy promotion, rape, forced sexual relations, humiliation and shaming, contraceptive refusal, threats of contraceptive sabotage, using better knowledge about contraceptives for manipulation and claiming that control is an expression of care, in the studied setting. For example, men’s PP acts, such as pressuring a woman to get pregnant, were continuous and persistent with a complete disregard of women’s motives to avoid a pregnancy.

When I finally got pregnant it was because of him, he told me ‘no’ he told me ‘you have to have a child, I want you to have a child, you have to have a child’ he nagged and nagged until I gave after. (Informant nr 6)

One of the subtle ways by which men exerted RC was framing it as a way of caring for their partners. This was expressed by men manipulating or withholding information on contraception to impose their own contraceptive choice to their partner’s. In one case, RC was not about imposing pregnancy, but by denying women’s agency to choose the contraceptive method to be used. Another manipulative tactic used by men to undermine women’s contraceptive self-efficacy was to question the woman’s ability to know whether she wanted a pregnancy or not.

Interviewer: In the beginning, when the two of you started to have a life together when you decided to live together, did you talk about family planning?

Respondent: Yes, but he always told me ‘no’, and that a woman isn’t with a man to use birth control, it’s for having children, but I said ‘no’, or like, in the beginning I didn’t want children right away, I wanted to have them later because I wanted to know him better, but no, he didn’t want to [use contraceptives]. (Informant nr 3)

Our interviews also showed that PP behaviors overlapped with emotional IPV. Men shamed and humiliated their partners who used contraception accusing them of infidelity. This situation often arose when women suspected their partners’ own infidelity and demanded them to use condoms. Our informants described how, having unprotected sex under those conditions was shameful in itself and perceived as forced sex. They also described how disagreements about contraceptives could result in rape.

[…] one day he came home in the morning and I remember I was always taking my contraceptives, my injections and yes, what he did was that he grabbed all those things the pills and he broke my family planning card. Then, he broke my pills and he hit me, right? He hit me eh…and then the accusations started and well as he had me there as if I was kidnapped, so I could not leave, right? And yeah sometimes he took, how do I say? He took me by force, and I did not like that, I did not like that […] (Informant nr 1)

### Overlapping RC types

**Fig. 3:**
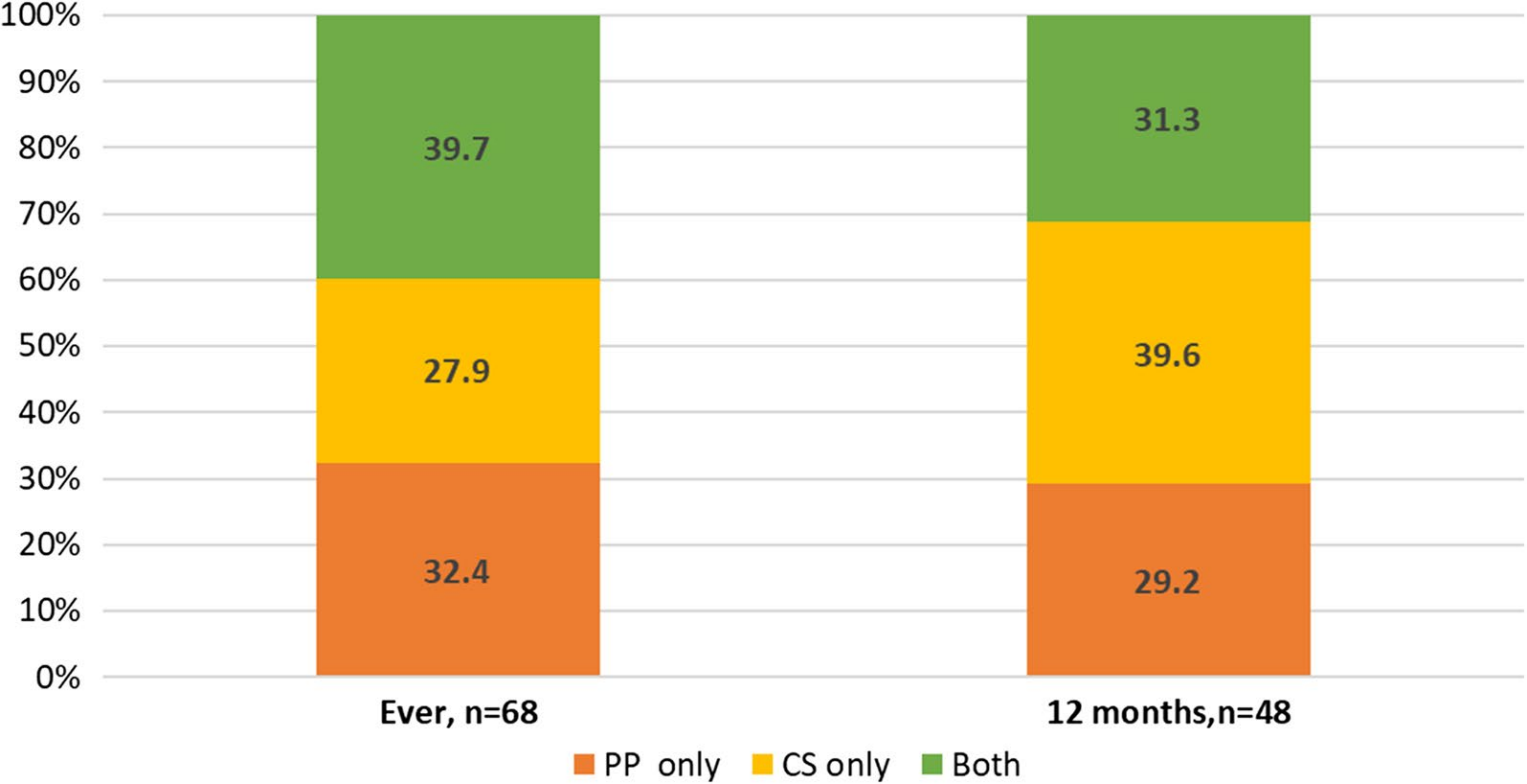
Overlapping between pregnancy promotion (PP), contraceptive sabotage (CS) ever, and last 12 months exposure

Overlapping RC types were common within the quantitative data. Four in ten women ever exposed to any form of RC were exposed to both PP and CS (three in ten among those reporting 12 months exposure to RC) (Fig. 3).

Our qualitative data also showed that women exposed to both PP and CS were the ones who were exposed to the most severe and explicit RC behaviors. In their narratives, PP was discussed more often than CS as well as the continuously nature of PP behaviors. For example, behaviors such as nagging the woman about having children, objecting when the woman was going to get her hormonal injection, or questioning that the woman should need any protection were discussed often. On the other hand, CS was described as happening occasionally. One of the informants told about how she repeatedly asked her partner if he had seen the card that she needed in order to get her free birth control at the HCC, he accused the children. When she found the card torn apart and confronted him, he admitted that he took it and broke it and told her that she “couldn’t decide by herself”. Her partner kept obstructing her intent to use birth control alongside his different PP acts.

### What factors were associated with RC?

Our multivariable quantitative analysis showed that after adjusting for possible confounders, women’s higher education was a protective factor against ever and 12 months of exposure to any RC behaviors by a current or former partner (Table 3). In addition, it was a protective factor against exposure to any CS in the twelve months before the survey. No other variables were significantly associated with ever RC, ever PP, 12 months RC, 12 months PP, or 12 months CS (Table 3).

**Table 3:** Adjusted prevalence rate ratios (APRR) of the association between women’s and their last partner’s demographic characteristics and ever exposure to reproductive coercion, n = 390

Variables	APRR (95% CI)^a^					
	Ever RC	Ever PP	Ever CS	12 months RC	12 months PP	12 months CS
Women’s education						
None	1.00	1.00	1.00	1.00	1.00	1.00
Primary school	0.40 (0.18–0.91)	0.58 (0.16–2.07)	0.50 (0.18–1.35)	0.39 (0.17–0.88)	0.82 (0.12–5.61)	0.46 (0.20–1.05)
Secondary school	0.28 (0.12–0.64)	0.34 (0.10–1.15)	0.40 (0.15–1.09)	0.24 (0.10–0.56)	0.38 (0.05–2.50)	0.28 (0.12–0.65)
University	0.35 (0.13–0.91)	0.32 (0.09–1.07)	0.53 (0.17–1.63)	0.22 (0.07–0.67)	0.37 (0.05–2.48)	0.28 (0.08–0.96)
Women’s working status						
Housewife	1.00	–	1.00	1.00	–	1.00
Student	0.43 (0.17–1.09)	–	0.13 (0.20–0.77)	1.02 (0.33–3.11)	–	0.21 (0.02–2.28)
Self-employed	0.73 (0.37–1.45)	–	0.54 (0.23–1.23)	0.51 (0.11–2.19)	–	0.30 (0.07–1.26)
Employed	0.82 (0.43–1.56)	–	0.92 (0.43–1.93)	0.50 (0.12–2.09)	–	0.85 (0.35–1.97)
Unemployed	0.87 (0.33–2.55)	–	0.93 (0.32–2.65)	1.00 (0.28–3.49)	–	1.31 (0.42–4.05)
Last partner’s education						
None	1.00	–	1.00	1.00	–	1.00
Primary school	1.26 (0.54–2.94)	–	1.08 (0.43–2.72)	1.07 (0.44–2.60)	–	0.80 (0.33–1.94)
Secondary school	1.08 (0.46–2.56)	–	0.60 (0.24–1.50)	0.90 (0.35–2.34)	–	0.62 (0.25–1.50)
University	0.85 (0.32–2.23)	–	0.34 (0.11–0.99)	0.78 (0.27–2.26)	–	0.32 (0.09–1.07)
Last partner’s working status						
Unemployed	–	–	1.00	–	–	–
Student	–	–	2.17 (0.81–5.83)	–	–	–
Employed	–	–	0.49 (0.27–0.88)	–	–	–

*RC* reproductive coercion, *PP* pregnancy promotion, *CS* contraceptive sabotage

^a^All models adjusted by the variables shown on the table

Women’s working status and their last partners’ education and working status were significantly associated with ever CS. Specifically, women whose last partner had university education had a 66% lower prevalence of ever CS than women whose partner had no education (APRR 0.34, 95% CI 0.11–0.99, Table 3). In addition, compared to women whose last partner was unemployed, women whose last partner was employed had a 51% lower prevalence of ever RC (APRR 0.49, 95% CI 0.27–0.88, Table 3). Finally, compared to women who were housewives, women who were studying had an 87% lower prevalence of ever CS (APRR 0.13, 95% CI 0.20–0.77).

### Women try to cope, but rarely succeed

Our qualitative data showed that women use different strategies to cope with RC and that they do so continuously, although they rarely succeeded in keeping control over their reproduction over a longer time span. The coping strategies implemented depended on the type of RC experienced, how the woman perceived it and how her partner framed it. It was also closely related to feelings as shame, fear, anger, and disappointment. We saw a broad spectrum of coping strategies in our data including: acceptance of RC, rationalization of RC, laughter, resistance, sterilization, planning for adoption, threatening the partner, hiding contraceptives, relying on God, buying hormonal emergency contraceptives (HEC), verbally objecting to RC, claiming to be in control and trying to control the partner.

Women who had no way of coping at the time of the exposure, coped during the interview. If the RC was framed as an act of care, the women did not cope at the time of exposure. During the interview, the coercive acts, framed and understood as care, were explained with acceptance, rationalization, and humor. One interviewee laughed a lot during the interview, she openly discussed her relation and emphasized how her husband took care of her, for example when they started having sexual relations. She explained how he took care of her by finishing outside of her, so she did not need to use hormones. They never talked about condoms at the time.

Interviewer: Ok, so initially you did not use any kind of protection, did you?

Respondent: No.

I: And how did you talk about having sexual relations like that, with that method?

R: With that method? He took care of me. I did not know anything about sexuality, nothing, my mother never talked to me about sexuality or that I would have my period, it was scary. Imagine that I was fifteen when I got my period and nothing only the people in the street told me […] (Informant nr 5)

Some informants explained how their partners “cared” for them were rather framed as rational since the women often lacked basic knowledge about reproduction and birth control while the men had that knowledge.

[…] he went to the pharmacy and bought it because first, he told me “condoms”, but I told him “no with that you will hurt me” I did not know anything about that, I was a virgin and all, so he came back with a small box and told me “you will get injected”. I got so nervous that I fainted because I didn’t know what he would give me, until afterward, he knew more than me, then when he came he told me it was a one-month injection to not get pregnant. Then they had already, they gave it to me when I was unconscious [laughing] I did not even notice it… (Informant nr 2)

The informant nr2 stated that she got upset, that she felt betrayed by her partner and that she would have preferred if he had discussed birth control with her first. But, then she continued by explaining that she understood that he did what he did to protect both of them and laughed about the fact that he had that kind of knowledge while she did not.

Equality and mutual decision making within the relationship was seen to be an ideal situation within the study population and had an impact on the coping strategies that women used during the interviews. Some interviewees claimed to be in control of their reproduction although the RC was part of their narrative. For instance, one informant stop using oral contraceptives and became pregnant after her partner demanded not to use them.

Interviewer (I): So, if you would have decided, at what age would you have had your first child?

Respondent (R): I was deciding.

I: Yes?

R: Because first I was looking after myself [by using contraceptives] and then I decided at what age I would have it [the child], or like, at the time I decided, “it’s fine, let’s have [a child]”

(Informant nr 6)

Not being able to control one’s reproduction and sharing stories of being pregnant although not really wanting it, created shame among the women. Some informants would make statements as “all children are a gift from God” or “it’s God’s decision if there is a child”. This further strengthens the interpretation that RC creates shame, and thus it could be easier to “blame” God instead of the person that one lives with.

The qualitative material shows that women are under a lot of pressure due to the lack of control that they impose over their reproduction. Planning to put an unborn and unwanted child for adoption, getting sterilized or a woman scaring her partner that she would abort the child if he made her pregnant against her will, are the more radical coping strategies used by the women exposed to RC in the studied setting.

Some informants knew that they would not be able to negotiate condom use with their partner, instead, they would make sure to have HEC at home to use after the sexual act.

## Discussion

Our main results show that one in ten women attending PHC services has been ever exposed to some form of reproductive control with pregnancy promotion being more common than contraceptive sabotage. However, of those exposed four in ten experienced both forms of RC. Women use different strategies to cope with RC but they are perceived as not useful.

The point prevalence of ever exposure to RC found our study is similar to figures reported from studies conducted in Cote d’Ivoire [6], Jordan [5], and the United States [2] but higher than data from India [7]. The later was surprising since we expected the point prevalence to be similar to other low-middle income settings.

The different instruments used to collect the data in both settings might explain the divergence in the prevalence described above. In the Indian study, the instrument measured RC from current husbands or in-laws whereas our study focused on identifying RC from current or former male partners [7]. This detail might explain why our prevalence is higher than the one reported in the Indian study. This highlights an important issue. Comparing RC data between countries is difficult when there is no standardized questionnaire measuring to RC as it is for other forms of violence against women, for example, the questionnaire used in the WHO multicounty study on women’s health and domestic violence [33]. Thus, efforts must be made to validate a comprehensive data collection instrument that can be used across different cultural settings.

A key finding in our study is that among women exposed to any form of RC (n = 116), 12 months exposure to only contraceptive sabotage is higher than ever exposure to the same type of violence (39.4% vs. 27.9% Fig. 2), whereas exposure to only PP remained close (29.2% vs. 32.4%). This finding shows how important it is to measure violence exposure in different time points. To be able to inform effective actions to protect women’s reproductive autonomy health care providers need to be able to detect not only previous but also current exposure to specific forms of RC.

Nicaraguan women in our sample were exposed to a broad range of RC that is in line with behaviors reported in other studies including men condemning birth control use [9], throwing away birth control pills/injections [3, 5, 6] or forcing women to have sex [1, 3, 17]. However, one new finding from our study was that men also framed RC as a way of caring for their partners. This finding can be discussed within the ambivalent sexism theory. Glick et al. [34] proposed that men could enact either hostile or benevolent sexism. Benevolent sexism is a form of masculinity that frames women as agentless weak individuals that need to be taken care of. Although benevolent sexism has been found to be protective against other forms of IPV [35], it is clear that its infantilization of women can also be used as an excuse to enact other more subtle forms of violence as shown in our study [34, 35].

In line with current literature elsewhere [1–3, 36], women’s increased educational levels were a protective factor against exposure to most forms of male reproductive coercion of women (ever or last twelve months). However, when stratifying by RC type (PP and CS), ever exposure to CS and last twelve months exposure to PP were not significantly associated with women’s education in spite of showing the same direction of association. This finding might be related to the lack of statistical power of our sample to detect a significant association in these sub-populations. More studies are needed to confirm this.

### Women try to cope with RC but rarely succeed

Our qualitative data showed that women used different strategies to cope with RC but rarely succeeded in preventing unintended pregnancies or regain their long-term fertility autonomy. This might be explained by the pervasive and continuous nature of men’s RC of women found in our study. Thus, even if women succeeded once in preventing an unintended pregnancy (i.e. by using emergency contraception), it is unlikely that they would have succeeded all the time. This is in line with previous quantitative studies in this setting showing that women who are exposed to different forms of intimate partner violence (emotional physical, sexual or controlling behaviors) have higher odds of using emergency contraception or reporting an unintended pregnancy [27, 37].

As described by Silverman and colleagues, one effective way to facilitate women’s effective coping with men’s reproductive coercion is to enable the availability and use of long-acting contraceptive methods that are less likely to be sabotaged by partners [17]. In addition, interventions challenging traditional forms of masculinities that highlight control of women as a key feature of manhood must be enacted consistently across settings. It is clear that without addressing this issue with the perpetrators, it will be difficult to decrease men’s reproductive coercion in this setting.

### Strengths and limitations

We argue that our results might underestimate the real prevalence of RC in Nicaragua. Our data were collected from women using urban PHC. The fact that these women were free to access PHC might signal that they have higher autonomy than those experiencing other forms of controlling behavior by their partners or those living in rural settings. Thus, in order to map the real prevalence of RC in Nicaragua, population-based studies must be conducted in both rural and urban settings.

Another limitation is that we did not measure how other forms of violence against women (emotional, physical, sexual, and controlling behavior) interacted with RC or how RC was associated with negative health outcomes such as unintended pregnancies, unsafe abortions, or women’s poor mental health. Further studies are needed in this setting to quantify these associations (if any). In addition, our qualitative data included only seven interviews with urban women. Thus, it is likely that the experiences and coping strategies of rural women would add more perspectives to our study.

Our study has significant strengths. Our MM design allowed us to have a comprehensive understanding not only on the different types of RC present in Nicaragua but to identify how women experience and try to cope with it. To the best of our knowledge, this is the first study in Latin America to do so on this topic.

## Conclusions

Our findings suggest that men’s RC of women attending PHC in Nicaragua is a common continuous phenomenon that can be enacted through explicit or subtle behaviors. Women in our small qualitative sample used different strategies to cope but failed to regain the long-term reproductive autonomy. Our findings can inform the development and implementation of new population-based studies which are needed to assess this phenomenon in a larger sample including both urban and rural settings. In addition, more qualitative studies are needed with men to explore how men understand and reflect upon men’s reproductive coercion of women. Given the negative consequences that men’s RC of women can have on women’s health and well-being. The Nicaraguan health system should train their staff to screen for RC and develop clinical guidelines allowing clinicians to provide effective contraceptive methods to their patients exposed to RC.

## Supplementary Information

**Additional file 1.** Qualitative interview guide.

## Data Availability

The data used in the analyses for the manuscript are not publicly available. However, they could be availed upon reasonable request by writing an e-mail to the corresponding author.

## Abbreviations

APRR: Adjusted prevalence rate ratio
CIs: Confidence intervals
CS: Contraceptive sabotage
HEC: Hormonal emergency contraceptives
IPV: Intimate partner violence
MM: Mixed-method
NGOs: Non-Governmental Organizations
PHC: Primary Health Care
PP: Pregnancy promotion
RC: Reproductive coercion
SD: Standard deviation
STI: Sexually transmitted infection
VAW: Violence Against Women
WHO: World Health Organization
