# Supplementary material for: Men’s reproductive coercion of women: prevalence, experiences, and coping strategies—a mixed method study in urban health facilities in León, Nicaragua
Source: BMC Womens Health. 2021 Aug 23;21:310. doi: 10.1186/s12905-021-01441-y (PMC8381564; doi:10.1186/s12905-021-01441-y)
Supplement: Supplementary file 1 — Additional file 1. Qualitative interview guide. [file 12905_2021_1441_MOESM1_ESM.docx]

Additional File 1. Qualitative interview guide

| **Topics** | **Questions** |
| --- | --- |
| Introduction and background | 1. Can you tell me a little bit about yourself? 2. How old are you? 3. Do you work? If yes ask, in what? 4. Do you have a partner now? 5. What is the last education level that you have attained? 6. What is your religion? 7. How many children do you have? |
| Power and reproductive decision making | 1. What is the right number of children to have? How so? 2. How did you decided to have the number of children that you currently have? Explore decision with current and former partners. 3. What happens if you and your partner/ex-partner disagree in the number of children to have?  - How so? - What did he do? How so? - What did you do? How so? |
| Contraceptive use | 1. What methods have you used to prevent pregnancy? How so? 2. How do you feel about using these methods? 3. At what age did you started using contraception? How so? 4. Who decided which method to use? How so? 5. What does your partner think about your current use of contraceptives methods? 6. Who decides on what method to use? How so? 7. Have you ever used emergency contraception? How so? 8. Who so you think have the responsibility to prevent a pregnancy? How so? |
| Reproductive coercion | 1. Have you ever wanted to use a contraceptive, but you could not? How so?  - Explore if she was coerced/forced to not use it  1. How did you deal with that situation? 2. Other women have told us that their partners have accused them of being unfaithful is they want to use contraception. What do you think about that?  - Has it happened to you? - How did you react?  1. Other women have told us that their partners have destroyed their contraceptive method (flushing it down the toilet, etc.) or that they have removed the condom during sex. What do you think about that?  - Has it happened to you? - How did you react? |
